# Lecture on the History and Treatment of Itch

**Published:** 1840-01-04

**Authors:** M. Cazenave


### CLINICAL LECTURE.

### HOSPITAL ST. LOUIS, (PARIS.)

### LECTURE ON THE HISTORY AND TREATMENT OF ITCH.

BY M. CAZENAVE.

[Reported for the Medical Examiner.]

*Wednesday, Oct.* 20*th.*—M. CAZENAVE commenced by observing, that although the itch was one of the diseases of the skin best known, and in general of easy diagnosis, still there were times when it presented great difficulties, which were the more dangerous inasmuch as the affection was extremely contagious. This is an affection essentially vesicular. Its origin and discovery are obscure. The psora of the ancients appears to have resembled it in some respects; but it is evident that they confounded other maladies with it, especially *impetigo.* The same remarks are true of the *scabies* of Celsus. More recently some authors have considered and described it as a pustular affection, while others have admitted a variety of this nature. This is an error that ought to be corrected before we proceed further; for whenever pustules exist with itch, it is always an accidental complication. Besides the contagion, which we have already noticed, the itch is characterized by vesicles ordinarily disseminated, accuminated, transparent at their summit, a little larger at their base, accompanied by an itching of variable intensity. Its ordinary seat is between the fingers, on the wrists, in the ham, the armpit, the groin, &c. In the flexions of the articulations we generally find it. It never attacks the face. Regarding the seat of itch, it is necessary to know that certain conditions and certain professions induce varieties and modifications, which ought not to be passed over in silence. Thus, blacksmiths, lockmakers, and dyers, rarely or never have it on their hands, because of the development and hardness of the teguments, directly the reverse prevailing among tailors and seamstresses; which last, by the way, furnish a majority of the cases of itch which enter this hospital. All ages are subject to the itch; it prevails at all seasons, though more particularly in summer,—in all climates, and in all social conditions. Never is it developed spontaneously, nor is it epidemic or endemic. The epoch of its incubation offers important varieties worthy of remark. Thus, among the aged, the period from the contagion to that of the invasion extends from two to three weeks, whilst in infants it is only from five to six days. In adults the incubation lasts, in spring and summer, from eight to ten days, and in the winter from fifteen to twenty.

*Symptoms.*—The vesicles of the itch first appear on that part of the skin which is thin, delicate, and provided with numerous lymphatics. The appearance is announced by an itching of variable intensity, which is increased by the heat of the bed, warm drinks, and exciting food. Vesicles now appear, which are slightly elevated,—these multiply, become acuminated, transparent at their summit, red in young subjects, and containing a serous and viscous liquid. Frequently, at the side of the vesicle, is a little furrow, of more or less extent. The itching increases progressively, and becomes most intense in the youngest subjects. Sometimes each vesicle is surrounded by a red areola, which is indicative of a more intense inflammation than properly belongs to itch.

If the disease is abandoned to itself, the eruption will progressively spread over the whole body, and be complicated with many other elementary forms, and particularly with impetigo, often with ecthyma, particularly in young and plethoric subjects, after a debauch. In fine, left to itself for a longer period, itch becomes complicated with other eruptive affections, as porrigo, &c. &c. The pruritus now becomes intense, insupportable; the patients scratch themselves, and tear the vesicles; the contained fluid is poured out, and the vesicles are now replaced by a number of small red points considerably inflamed.

Itch is never dangerous, however old or neglected it may be; and the internal inflammations and eruptions which accompany it are always purely accidental complications.

If, however, instead of permitting the disease to pursue uninterruptedly its course, we early attack it with suitable remedies, the vesicles fall, there is a slight desquamation, the eruption does not reappear, and the disease is cured.

*Causes.*—At the present time the proximate cause of itch is known to be an insect, called *acarus scabei.* The existence of this animal has been the subject of numerous discussions. Acknowledged at first by Avenzoar, and afterwards by Ingrassias and Joubert, the acarus was described at length by Mousset, and designed after nature by Hamptmann. François Redi presented a detailed account of it in the seventeenth century, both of its development, and of the manner in which it produced the itch. Finally, the acarus was classified in natural history by Linnæus, De Geer, Fabricius, and Latreille. Forgotten by a great number of physicians, others doubting its existence, in 1812, M. Galés, formerly pharmacien to this hospital, revived the subject by a series of experiments, which, for a while, appeared to set all doubts at rest. He described its physical characters, its habits, &c.

According to M. Galés, the insect was found in the vesicle itself. M. Biett repeated these experiments, but without the same results. In the years 1818 and 1819 he made numerous researches with Amici's horizontal microscope, but without success. Similar investigations were made since 1813, in the service of M. Alibert, without greater success, as well as in Italy, by numerous physicians, and by M. Lugol here. Notwithstanding the authority of Morgagni, the existence of the insect was again involved in doubt, the experience of M. Galés proving nothing, not having been verified by other observers. Such was the opinion which prevailed in France up to 1834, when a student of medicine, M. Renucci, demonstrated at this hospital the proper method of finding the acarus, having seen very frequently the women in his native country—Corsica—extract the insect of the itch, and having frequently extracted it himself. It is not in the vesicle that we are to look for it, but in the furrow, which runs from it, and which the insect forms under the epidermis. You now understand how many attempts were made before a successful issue; and you may remember that Mousset, among others, announced that the insect was not found in the pustules, but alongside; an important circumstance, upon which other pathologists have insisted, and which has been clearly illustrated in the beautiful work of Mr. J. Adams. It is now clearly demonstrated that M. Galés was deceived, as the acarus not only does not occupy the vesicle, but neither does it resemble the mite of cheese, to which animal this observer compared it. M. Raspail, who has described the acarus very well, and given some excellent plates of it, has proposed to call it *sarcoptes hominis.*

The difficulty of finding the acarus has induced many to believe that it does not invariably occur, and to ask whether its presence is not accidental, as that of *pediculi* in certain cases of porrigo. To this a negative answer may be made, for it is now proved that the presence of the acarus is indispensable to the production of itch. Such, gentlemen, is the proximate cause of itch. The predisposing cause, youth and adult age, the male sex, a sanguine temperament, such occupations as keep the hands perpetually moist, and a neglect of cleanliness.

Can itch be communicated to the human subject from animals?

Some years since, a number of men, employed in attending the camels at the Garden of Plants, were admitted into this hospital. The animals were severely affected with the disease on their arrival from Africa. A prosector of the Museum stated that the insect, in this case, belonged to another species of insect; but this opinion was not verified by the researches of MM. Biett and Schedel. The error arose from the fact that dogs and other animals are liable to other diseases of the skin besides itch.

*Diagnosis.*—Easy to recognise in the majority of cases, the itch becomes, sometimes, very difficult to distinguish from some other affections of the skin entirely different, and, above all, not contagious.

Itch cannot be confounded with the erythematous affections. Amongst the vesiculous affections, it can be confounded with *eczema*, and particularly *eczema simplex.* But here the vesicles, when they even exist at the internal portion of the arm and fingers, are flattened, and not acuminated, as in the itch. They are confluent, and not discreet, and are as often seen upon the back of the hand as upon the flexions. The pruritus of eczema is a kind of general smarting, differing from the exacerbations which characterize the itch. All doubt, in fine, will be dissipated, on proving the existence of the acarus.

*Herpes* can be distinguished from itch by its coming in groups. Of the *papulous* diseases prurigo is the only one that can be confounded with itch. Independent of the primitive characters, which one can always recognize, the ordinary seat of prurigo is the back, the shoulders, and the limbs in the line of extension. In prurigo, the papules present, on the summit, a blackish crust, which is not the case of itch. In fine, the itching in prurigo is very disagreeable, and not comparable to that of itch.

It is impossible to confound the itch, as I have already said, with any of the pustulous affections.

In spite of all the characteristic signs which we have attempted to give for the diagnosis of itch, it sometimes happens that it becomes exceedingly difficult to distinguish it. It is better in such cases to abstain from making any diagnosis until further circumstances assure you that you are correct.

*Prognosis.*—This is never grave, but without treatment this disease is never cured. Can it be transformed into other affections of the skin, as lichen, prúrigo, impetigo, &c.? Certainly not; but they can complicate it. One thing you ought to know, and which is by no means rare; it is, that in patients that have suffered from itch, a vesicular affection occurs periodically every year, especially during hot weather, and which may be mistaken for itch. This affection is not absolutely the itch, but a kind of sequel, and which is not contagious.

*Treatment.*—The itch is readily cured by a number of remedies. The different medicines have, however, one common end—the modification of the cutaneous affection, or its destruction. In a word, the treatment of itch is purely locally empirical. Formerly, we associated with the local treatment a depurative treatment, which principally consists in bleeding, purgatives, demulcents, &c. This has been useless, and is now abandoned. In some cases, however, it becomes necessary to resort to general means, as when it is complicated with phlethora, or when there is cutaneous inflammation. If, on the other hand, the patient is enfeebled, you must resort to tonics, and other strengthening measures. If neither of these conditions obtain, you must confine yourselves simply to local means. So numerous are the local means, that in mentioning them we must confine ourselves to those which are dangerous, and to those which are confessedly efficacious.

Among the ointmemts, those made with the mercurial preparations, as citrine ointment, mercurial ointment, &c. &c., ought not so to be used, for fear of their producing eczema rubrum, and retarding the cure; they produce nearly constantly swelling of the salivary glands, salivation, and sometimes even glossitis.

The ointment of hellebore (pommade d'ellebore) has succeeded in the hands of M. Biett, without any accidents supervening.

The ointment of Helmerich, modified by M. Biett, is one of the best and most efficacious means we can apply. Thus:

℞.—Sulphat. sub. ℥ii.

Calc. subcarb. ℥j.

Axungiæ. ℥viij.

Rub in a ℥ss every morning and evening.

The method of Pyhorrel succeeds very well in slight cases. It consists in frictions of the sulphate of lime, simply powdered, and mixed with a small quantity of olive oil at the moment of using it. A half ounce of the sulphate at each friction, which should be twice a day.

*The Lotions of Dupuytren* are useful for those patients who do not wish to rub themselves.

℞.—Potass. sulph. ℥iv.
Acid sulph. ℥ss.
Aq. Ojss.
M.

The patients bathe themselves twice a day with this. It has the double objection of having a bad odor and being irritant.

The *soap lotions* may be used among children. Sulphurous baths possess the same objections as the baths, and produce irritation, and particularly eczema rubrum. The alkaline ointment, composed of ʒij of carb. potass. to the ℥j of lard, has been recommended by many. Cauterization (la methode ectrotique) offers no advantages, except at the very commencement of the disease, and then it will, very probably, produce irritation and even phlegmonous inflammation, which will prolong the duration of the itch itself.

In some of the old books, you will find decoctions made with aromatic plants recommended. Experience does not, however, place their reputation, as remedial means, very high. If they are beneficial at all, it is through means of the essential oils they contain, and, consequently, these infusions would be more efficacious.

Lastly, it is particularly necessary to disinfect the clothing with care, especially those with linen, by exposing them to the action of sulphurous acid gas. Relapses are to be prevented, and great care enjoined upon the patient.

